# Molecularly Targeted Therapy of Human Hepatocellular Carcinoma Xenografts with Radio-iodinated Anti-VEGFR2 Murine-Human Chimeric Fab

**DOI:** 10.1038/srep10660

**Published:** 2015-05-29

**Authors:** Jianfei Huang, Qi Tang, Changjun Wang, Huixin Yu, Zhenqing Feng, Jin Zhu

**Affiliations:** 1 Key Laboratory of Antibody Technique, Ministry of Health, Nanjing Medical University, Nanjing, Jiangsu 210029, China; 2 Department of Pathology, Affiliated Hospital of Nantong University. Nantong, Jiangsu 226001, China; 3 Huadong Medical Institute of Biotechniques, Nanjing, Jiangsu 210002, China; 4 Key Laboratory of Nuclear Medicine, Ministry of Health, Jiangsu Institute of Nuclear Medicine, Wuxi, Jiangsu 214063, China; 5 Jiangsu Key Lab of Cancer Biomarkers, Prevention & Treatment, Cancer Center, Nanjing Medical University, Nanjing, Jiangsu 210029, China

## Abstract

Vascular endothelial growth factor receptor 2 (VEGFR2) is traditionally regarded as an important therapeutic target in a wide variety of malignancies, such as hepatocellular carcinoma (HCC). We previously generated a murine-human anti-VEGFR2 chimeric Fab (cFab), named FA8H1, which has the potential to treat VEGFR2-overexpressing solid tumors. Here, we investigated whether FA8H1 can be used as a carrier in molecularly targeted therapy in HCC xenograft models. FA8H1 was labeled with ^131^I, and two HCC xenograft models were generated using BEL-7402 (high VEGFR2-expressing) and SMMC-7721 (low VEGFR2-expressing) cells, which were selected from five HCC cell lines. The biodistribution of ^131^I-FA8H1 was determined in both models by Single-Photon Emission Computed Tomography and therapeutic effects were monitored in nude mice bearing BEL-7402 xenografts. Finally, we determined the involvement of necrosis and apoptotic pathways in treated mice using immunohistochemistry. ^131^I-FA8H1 levels were dramatically reduced in blood and other viscera. The therapeutic effect of ^131^I-labeled FA8H1 in the BEL-7402 model was significantly better than that by ^131^I and FA8H1 alone. We observed extensive necrosis in the treated tumors, and both FasL and caspase 3 were up-regulated. Thus, ^131^I-anti-VEGFR2 cFab has the potential to be used for molecularly targeted treatment of HCC overexpressing VEGFR2.

Human liver cancer, particularly hepatocellular carcinoma (HCC), is the sixth most common neoplasm worldwide and the third highest cause of cancer deaths worldwide^1^. Most HCC patients are diagnosed at an advanced stage, when traditional treatments are not effective^2^,^3^. Despite the advances in surgery, liver transplantation and systemic chemotherapy, the survival rate of HCC patients has not improved much over recent decades^2^,^3^. Monoclonal antibodies (mAbs) can be used for molecular imaging as well as cancer-specific vehicles to deliver therapy to the tumor site^4^,^5^. However, the use of murine mAbs is limited in the clinic because of their high immunogenicity, large molecular weight, and the risk of human anti-mouse antibody (HAMA) responses^6^,^7^. Murine-human chimeric Fab (cFab) was prepared from a murine antibody. cFab offers several advantages over whole murine IgG: first, molecular weight of murine-human chimeric Fab (cFab) is approximately 50 kDa and only one-third of the size of full-length IgG. Secondly, cFab reduces the HAMAs responses as it is prepared by recombining whole murine variable regions with human constant regions^8^,^9^,^10^,^11^,^12^.

Many cFabs have been studied under pre-clinical or clinical development, and have become one of the most prolific drug classes in oncology^13^,^14^,^15^. We previously produced a high-affinity murine anti-vascular endothelial growth factor receptor 2 (VEGFR2) mAb (A8H1) using mouse hybridoma technology^16^, and constructed anti-VEGFR2-cFab (FA8H1) containing the variable region from A8H1 and the constant region from human IgG. The chimeric Fab maintained the specificity for the VEGFR2 antigen^17^.

VEGFR2 plays an important role in angiogenesis in a wide variety of malignancies^18^,^19^,^20^, such as HCC^21^. Our previous study confirmed the prognostic significance of VEGFR2 overexpression in HCC^22^. VEGFR2 has also been investigated as an anticancer target^23^,^24^,^25^. In fact, one VEGFR2 mAb, ramucirumab (IMC-1121B) is currently being tested in the treatment of human cancer^26^,^27^.

Radioimmunotherapy (RAIT) involves the use of mAbs in combination with therapeutic radionuclides, which have been increasingly used in the clinical setting^28^,^29^. For example, both yttrium-90-ibritumomab tiuxetan (Zevalin) and ^131^I-labeled Tositumomab (Bexxar) are FDA-approved to treat non-Hodgkin’s lymphoma (NHL)^30^,^31^,^32^.

In this study, we investigated the therapeutic efficacy of radioiodinated anti-VEGFR2-cFab (FA8H1) on human HCC xenografts. We determined the biodistribution of ^131^I-labeled FA8H1 and its therapeutic effects *in vivo*, and the hematological toxicities from the iodinated antibody were assessed. Finally, we determined the involvement of necrosis and apoptotic pathways in the therapeutic effects of ^131^I-labeled FA8H1.

## Materials and methods

### Cell Lines and Culture

Five HCC cell lines, BEL-7402, QCY-7701, SMMC-7721, HepG-2 and SK-HEP-1, were purchased from the cell bank of the Chinese Academy of Science in Shanghai, China. They were cultured in DMEM medium (Gibco, Invitrogen, USA) containing 10% fetal bovine serum (FBS) at 37 °C and 5% CO_2_.

### Fluorescence-Activated Cell Sorting (FACS)

HCC cell lines were treated with FA8H1 at 20 μg/ml overnight at 4 °C. After washing with phosphate-buffered saline (PBS), they were incubated with 0.01% rhodamine-conjugated anti-human Fab antibody (Rockland, USA). Cells were then centrifuged and washed, and subsequently analyzed on a BD FACS Calibur using the Cell Quest program. Background staining was obtained by replacement of the primary antibody with the sonicated supernatant of *E. coli* TOP10F´^17^. And the experiment was repeated by displacement of the primary antibody with PBS as a negative control which was consistent with the control using the sonicated bacterial supernatant.

### Radiolabeling of Anti-VEGFR2-cFab

Murine-human chimeric anti-VEGFR2-Fab (FA8H1) was previously generated in our laboratory^17^. The chloramine-T method^33^ was used to label the antibody with ^131^I. Briefly, 2.0 mCi (74 MBq) of ^131^I (Gaotong, Chengdu, China), 100 μg of FA8H1, and 200 μL of 0.2 M phosphate buffer (pH 8.0) were added to vials coated with 50 μg Iodogen (Sigma-Aldrich, St. Louis, MO) and incubated for 10 minutes at room temperature. Then the mixture was separated from free iodide by passing over an equilibrated PD-10 desalting column (GE, Niskayuna, NY, USA). The labeling efficiency was determined in a Perkin Elmer 1470 Automatic Gamma counter (Fremont, CA, USA). The radiochemical purity (RCP) of ^131^I-FA8H1 was assessed by a trichloroacetic acid (TCA) assay, as described elsewhere^33^,^34^, and the stability of ^131^I -FA8H1 *in vitro* was determined by incubating of the ^131^I-FA8H1 in murine blood with heparin at 37 °C for 24 h.

### Immunoreactivity of Radiolabeled Anti-VEGFR2-cFab

HCC cell lines were harvested by scraping using TrypLE Express (Invitrogen, USA) and washed with PBS (pH 7.4). A total of 2 × 10^6^ cells were re-suspended in 1 ml PBS (pH 7.4) containing 0.2% BSA, and incubated with 10 μg/ml ^131^I-FA8H1 in a 37 °C water bath for 1 h. Cells were washed twice and spun at 2,000 rpm for 10 min. The radioactivity of the pellets was then read by a gamma reader. Same number of cells were incubated with the presence of a 200-fold molar excess of ^131^I-labelled unrelated cFab (human-murine chimeric antibody against protective antigen of Bacillus anthracis, which was prepared in our lab)^35^ to obtain the nonspecific binding as the blank (NSB, nonspecific binding). The assay was repeated three times.

### Human HCC Xenograft Model

Two HCC cell lines with the highest and lowest VEGFR2 expression among the five cell lines were used to develop two human liver cancer xenografts. Six-week-old female athymic nude (nu/nu) mice (National Rodent Laboratory Animal Resources, Shanghai Branch) were injected with 2 × 10^6^ HCC cells in the left hind limb per 100 μl DMEM medium without FBS by subcutaneous implantation. For all studies, mice had access to laboratory food and water *ad libitum*. Drinking water contained 0.1% potassium iodide to block thyroid uptake of iodine. All experimental protocols were approved by the Research Ethics Committee of the Nanjing Medical University and Jiangsu Institute of Nuclear Medicine, China. All experimental procedures involving animals and their care were carried out in accordance with the Nanjing Medical University and Jiangsu Institute of Nuclear Medicine for Animal Experimentation and the National Institutes of Health Guide for the Care and Use of Laboratory Animals.

### Biodistribution Studies

Total 36 tumor-bearing nude mice with high VEGFR2 expression were randomly divided into six groups (n = 6/group) when tumors had reached 0.5–0.8 cm in diameter as measured by external caliper measurement. Animals were injected with 10 μCi ^131^I-FA8H1 through the tail vein under light inhalation anesthesia. Then whole-body images of each mouse were acquired at 6, 12, 24 and 48 h post-injection by Single-Photon Emission Computed Tomography (SPECT, Skylight; Philip, Amsterdam, the Netherlands). The mice were sacrificed at 1, 6, 12, 24, 48, and 72 h, and tumors and tissues (blood, brain, heart, liver, spleen, kidney, and lung) were removed for measurement of ^131^I-FA8H1 accumulation using a gamma counter. The results were calculated as a percentage of the injected dose per gram of tissue (%ID/g) [34].

### Imaging studies

Both (n = 6/group) HCC xenograft models, were used on imaging study when the tumor’s dimension achieved 1.0 cm in size. Prior to the imaging session, mice were anaesthetised with 13 mg/kg xylazine and 87 mg/kg ketamine. In brief, each mouse was injected with 200 μCi ^131^I-FA8H1 through the mouse tail vein. Whole-body images of each mouse were acquired at 6, 12, 24 and 48 h post-injection by SPECT.

### Radioimmunotherapy with ^131^I-FA8H1

A total of 48 mice injected with BEL-7402 cells were divided into six groups when the average diameter of the tumors reached 0.3 cm. Three groups of mice (8 mice per a group) were administered varying amounts of ^131^I-FA8H1 (100, 200, and 300 μCi) via the tail vein at 2-week intervals under light inhalation anesthesia. The other three groups of mice (8 mice per a group) served as controls, receiving 300 μCi ^131^I along with 300 μg unlabeled FA8H1 and PBS in the same volume. Tumor volumes were measured once a week for 42 days and calculated according to the following equation: tumor volume (TV) = π/6 × a × b2, where a is the longest diameter, and b is the shortest diameter, as we described elsewhere^36^. These animals were sequentially killed by CO_2_ asphyxiation. Tumors were removed, weighed, and stored for further use.

### Hematoxylin-Eosin (H&E) Staining

Tumor were formalin-fixed and paraffin-embedded. All sections were cut to 4 μm on a manual rotary microtome (Leica RM2235, Germany). The sections for H&E were stained by an Automated Slide Stainer (Leica ST5020, Germany) and cover slipped with a Robotic Coverslipper (Leica CV5030, Germany). The extent of tumor necrosis was divided into four grades, including without or including illegibility necrosis (-), spotty necrosis (+), small schistic necrosis (++) and big schistic necrosis (+++).

### Immunohistochemistry (IHC) and Scoring

Deparaffinized sections (4 μm-thick) from paraffin blocks were separately stained as we described previously^37^,^38^ using the following primary antibodies: rabbit polyclonal to Fas Ligand (1:1000, ab15285, Abcam, USA) and rabbit polyclonal to Caspase 3 (1:1000, ab4051, Abcam, USA). Secondary antibody was Envision goat anti-rabbit HRP (DAKO, USA). The evaluation of the immunostaining of these proteins was blinded to the pathologists (Huang J and Feng Z) by an independent observation simultaneous design. The sum of the percentages and intensity scores was used as the final staining score, as described previously^22^.

### Statistical Analysis

Using the STATA V.9.0 Statistics package(version 9.0; Stata Corp, College Station, TX, USA), the analysis of variance (ANOVA) and the least significant difference (LSD) method were used to assess changes in tumor weight and tumor volume. Mean ± SD was calculated for each group. The degree of tumor necrosis and the disparate expression of FasL or caspase 3 in tumor tissues were examined using the Kruskal-Willis test. For all analyses, data were analyzed using STATA, and a *P* value <0.05 was considered to be statistically significant.

## Results

### Native VEGFR2 Antigen Expression in HCC Cell Lines

**Figure 1:**
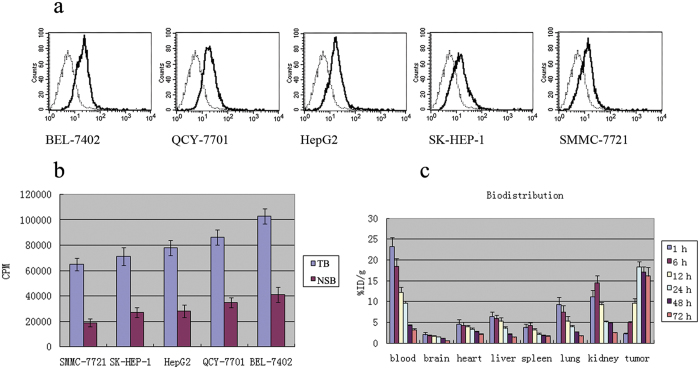
**a**: FACS analysis. FA8H1 binding in HCC cell lines is shown as a bold line. Background staining (shown in dashed line) was obtained using sonicated precipitation of *E. coli TOP10F′* as a negative control. **b**: Specific binding assay of ^131^I-FA8H1 in five HCC cell lines. Cells were suspended in 0.2% BSA-PBS and split into two groups. One group was incubated with ^131^I-FA8H1 (designated as TB, total binding) at a final concentration of 10 μg/ml for 1 h in a 37 °C water-bath. Cells were washed twice and spun at 3,000 rpm for 10 min, then were read with a gamma reader and shown as counts per minute (CPM). Another group was incubated with the presence of a 200-fold molar excess of ^131^I-labelled unlabeled cFab to determine the nonspecific binding as the blank (NSB, nonspecific binding). The various specific bindings of ^131^I-FA8H1 in these five cell lines from lowest to highest were SMMC-7721, SK-HEP-1, HepG2, QCY-7701, and BEL-7402. **c**: Serial biodistribution of ^131^l-labeled anti-VEGFR2 cFab in nude mice bearing BEL-7402 (high expression of VEGFR2) tumors (n = 6 at each time point). At 24 h, 18.31% (±1.31) of injected activity was found in the tumors at the peak, whereas blood activity had already fallen to 9.57% (±0.46) and levels in other normal tissues were even lower. At 48 h, there was good retention of activity within tumors at 17.05% (±1.28) of injected activity, whereas blood activity was further fallen to 4.28% (±0.22).

To compare the expression of native VEGFR2 in HCC cell lines, FACS was performed to analyze the five human HCC cell lines. All lines showed various degrees of binding with anti-VEGFR2 antibody (A8H1) in the following order, from highest to lowest based on the geometric mean fluorescence: BEL-7402, QCY-7701, HepG2, SK-HEP-1 and SMMC-7721 (Fig. 1a).

### Binding and Immunoreactivity of Radioiodinated Antibody

The radiochemical purity of the labeled antibody was found to be >95.5%, and the specific activity of ^131^I-FA8H1 ranged from approximately 1.0 to 1.5 mCi/mg. Radiopharmaceutical quality control was confirmed by layer chromatography. The amount of free nuclide in blood fell from 98 to 97.5% in 24 h at 37 °C. Specific binding between ^131^I-FA8H1 and native VEGFR2 in HCC cells was confirmed with a gamma counter by measuring binding between the cells and ^131^I-FA8H1, representing the total binding, versus a radiolabeled unrelated cFab, representing non-specific binding (Fig. 1b). The specific binding of the FA8H1 antibody was substantial after comparing total binding and non-specific binding, especially when the fact that a 200-fold molar excess of radiolabeled non-specific antibody was used is taken into account. These results were consistent with the data obtained by flow cytometry analysis and previous western blotting data for VEGFR2 in HCC cell lines^22^. BEL-7402 and SMMC-7721 had the highest and lowest native VEGFR2 expression of the five HCC cell lines, respectively, and were thus chosen as the suitable cell lines for subsequent experiments.

### Biodistribution Studies *in Vivo*

As shown in Fig. 1c, radioactivity levels were greatly reduced in blood and other viscera, and elevated to the highest levels (18.31% (±1.31)) in tumors at 24 h. With time, blood activity and levels (9.57% (±0.46)) in other normal tissues presented discontinuous and weaker expression, while good retention of activity in tumors could be detected at 72 h (F = 5.33, *P* < 0.0001). The biological half-life of the ^131^I-FA8H1 in the BEL-7402 xenograft was estimated to be 120 h from this portion of the decay curve.

### Effect of ^131^I-FA8H1 *in vivo* imaging on HCC model

**Figure 2:**
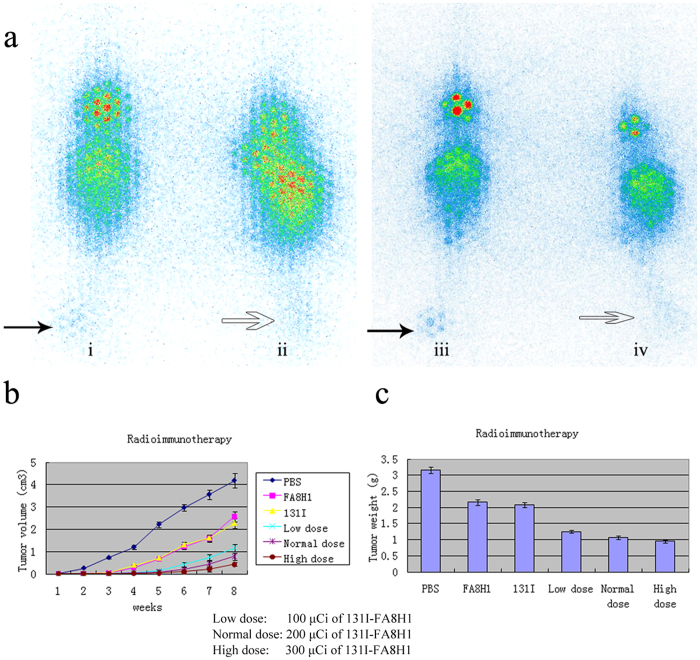
**a**: ^131^I-FA8H1 (200 μCi ) was injected and imaging was performed using a SPECT at various periods to observe *in vivo* radiolocalization. Solid and hollow black arrows indicate tumors: i, nude mice bearing BEL-7402 (high expression of VEGFR2) at 12 h post-injection; ii, nude mice bearing SMMC-7721 (low expression of VEGFR2) at 12 h post-injection; iii nude mice bearing BEL-7402 at 24 h post-injection; iv, nude mice bearing SMMC-7721 at 24 h post-injection. **b** and **c**: Growth curves of tumor volume (b) and final tumor weight (c) for each nude mice-bearing BEL-7402 group (n = 8), indicate a clear relationship between administered activity and response, although significant variability is seen within each group.

SPECT images were acquired for individual mice bearing BEL-7402 HCC model and SMMC-7721 HCC model, which separately had high and low VEGFR2 expression levels. Since injected with the ^131^I-FA8H1, the tumor in the left hindlimb of these mice was visible within 6 hours after tracer injection in the two kinds of tumor models. As the metabolism of these animals, the image of brain and other viscera get more and more down-expression along with time passing. At 24 h post-injection, the tumor in the higher VEGFR2 expression of BEL-7402 HCC xenograft was easily seen, and the representative patterns were listed in Fig. 2a, which supported the consequence of biodistribution *in vivo* study in some degree.

### Changes in Tumor Volume and Weight in the HCC Xenografts

To reduce the number of animals used, we focused this experiment on mice bearing BEL-7402 tumors which showed the highest expression level of VEGFR2 among the five HCC cell lines tested. As shown in Fig. 2b,c, no anti-tumor activity was observed in the PBS control group. Prominent inhibition of tumor volume and weight with high dose of 300 μCi ^131^I-FA8H1 was observed, and remarkable growth-inhibitory effects could be seen in the 300 μg FA8H1 alone group or the 300 μCi ^131^I alone group as compared with the PBS control group. No significant difference was found in tumor growth between the 300 μg FA8H1 alone group and the 300 μCi ^131^I group. Furthermore, the anti-tumor result was significantly enhanced with the elevated dose of ^131^I-FA8H1 (100, 200, and 300 μCi) during the process of tumor growth (F = 9.88, *P* < 0.001, tumor volume; F = 701.45, *P* < 0.001, tumor weight), the detail could be seen in the Fig. 2b,c.

### Histopathologic Tumor Necrosis in HCC Tissue

**Figure 3:**
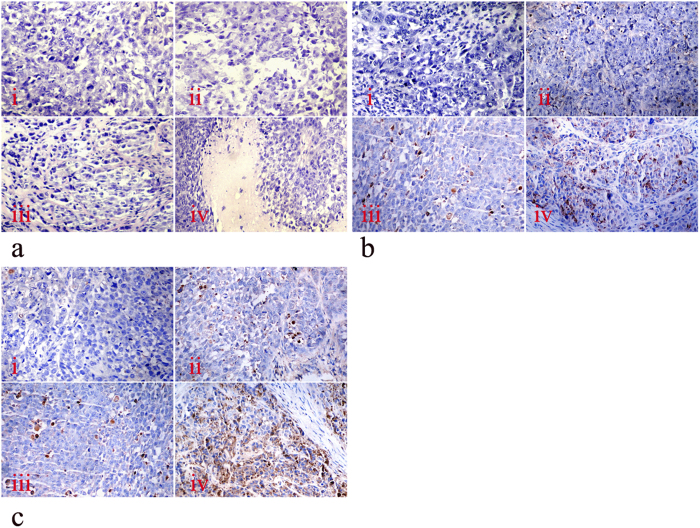
**a**: Histologic changes after RAIT treatment with ^131^I-FA8H1 or control (hematoxylin-eosin staining, 200-fold magnification) in BEL-7402 xenograft: i, HCC tissue in PBS control group; ii, HCC tissue in FA8H1 group with fibrinoid necrosis; iii, HCC tissue in ^131^I group with minor coagulative necrosis; iv, HCC tissue in ^131^I-FA8H1 group with large area of coagulative necrosis. **b** and **c**: Immunohistochemical staining of FasL (b) and caspase 3 (c) in HCC tissues after RAIT in nude mice bearing BEL-7402 with ^131^I-FA8H1 or control (200-fold magnification): i, HCC tissue in PBS control group was negative for FasL and caspase 3; ii, HCC tissue in FA8H1 group, FasL and caspase 3 was detected in a small number of cancer cells; iii, HCC tissue in ^131^I group with few cancer cells was positive for FasL and caspase 3; iv, HCC tissue in ^131^I-FA8H1 group with high FasL and caspase 3 expression.

The morphological changes characteristic of tumor necrosis could be easily observed using H-E staining. As shown in Table 1 and Fig. 3a, the category and the extent of necrosis were disparate with distinct therapeutics. Although necrosis was not significant in the PBS group, fibrinoid necrosis was identified in the FA8H1 alone group. Moreover, coagulative necrosis presented to a minor extent in the ^131^I group and in large areas in the three ^131^I-FA8H1 groups. Moreover, we observed different levels of radionecrosis resulting from the various doses of ^131^I-FA8H1 treated in the three groups with obvious statistical difference (χ^2^ = 33.253, *P* < 0.0001).

### Effects of Treatment through Apoptotic Pathways

The expression of two apoptosis indicator proteins, FasL and caspase 3, in the tumor tissues was evaluated by immunohistochemical staining (Fig. 3a,b). Compared with the HCC tissue of the PBS group, FasL and caspase 3 expressions in the other five groups were both increased, especially in the three ^131^I-FA8H1 groups (*P* < 0.001; Table 2).

## Discussion

In targeted therapy to human malignant tumors, the ideal therapeutic drug only affects tumor cells, having no influence on normal cell proliferation, and does not cause toxicity *in vivo*^39^. VEGFR2 was initially considered to be one of the most important targets in anticancer treatment, and its targeting could meet the above requirements^19^,^40^. Our previous work demonstrated that the VEGFR2 expression in liver cancer was much higher than in normal liver tissues, and this high VEGFR2 expression was associated with the poor outcome of these patients^22^. Moreover, we established an active murine-human chimeric Fab antibody (FA8H1), which is added to the list of potential therapeutic agents against solid tumors overexpressing VEGFR2^17^.

In the current research, the chimeric Fab antibody was radioiodinated with ^131^I to investigate its therapeutic efficacy. A minimal biodistribution study was performed for this experiment prior to the therapy studies to confirm selective tumor localization and retention. The biodistribution study showed that tumors with high VEGFR2 expression could be clearly detected at appropriate time points and that the retention of the ^131^I-labeled chimeric antibody was limited in other normal organs. This result was in agreement with previous reports of anti-VEGFR2 antibody as an imaging agent in an *in vivo* tumor model^41^.

The therapeutic effect of the ^131^I-FA8H1 was confirmed in this study showed that its administration led to a reduction in tumor weight and volume; thus, ^131^I-FA8H1 may be a promising tool to treat cancer *in vivo*. Our findings provided evidence that the function of ^131^I-labeled chimeric anti-VEGFR2 Fab *in vivo* relies heavily on the recognition of VEGFR2 protein.

We also investigated the degree of necrosis under the targeted therapy using the ^131^I-labeled antibody. Apoptosis was confirmed by monitoring FasL and caspase 3 levels. These data revealed the necrosis and apoptosis pathways were activated by ^131^I or ^131^I-FA8H1. Then, the effect of treatment with the ^131^I-labeled chimeric anti-VEGFR2 Fab was determined. To our knowledge, these observations were seldom made in previous studies^19^,^42^,^43^ that reported the anti-tumor role of radionuclide labeled mAbs.

There were some limitations of this study. First, our results lack clues regarding the detailed molecular mechanism involved in the binding of the anti-VEGFR2 antibody and VEGFR2 antigen on the tumor cell surface. Moreover, the side-effects and toxicity issues were ignored in our present investigation. Furthermore, following observations of neutralizing effectiveness of FA8H1 in the HCC animal model, we did not perform further experiments to verify and expand the neutralizing antibody. In addition, the biodistribution and RAIT were studied in mice, but it remains to be determined whether those conclusions will also apply to human patients with HCC. Also in this investigation, we mainly focused on the cancer cells in the tumors; since VEGFR2 plays an important role in the formation of tumor angiogenesis, we did not examine the changes in new blood vessels formation in tumors resulting from the targeted treatment. Finally, it remains to be determined if FA8H1 is specific to HCC or whether it plays a similar functional role in other human malignancies.

Since we have established that VEGFR2 expression is low in adult human liver^22^,^44^, and that HCC can be treated in a mice model via the caudal vein, we believe ^131^I- FA8H1 has clinical translational potential in the targeted treatment of human HCC with high expression of VEGFR2. On this basis, this study clearly indicates that ^131^I-labeled chimeric anti-VEGFR2 Fab could be used as a therapeutic drug for RAIT through targeting VEGFR2 antigen with anti-apoptotic and necrosis effects in HCC xenograft model in mice. Thus further studies are needed to determine the mechanisms involved in the neutralizing effect of FA8H1 on HCC tumorigenesis.

## Additional Information

**How to cite this article**: Huang, J. *et al.* Molecularly Targeted Therapy of Human Hepatocellular Carcinoma Xenografts with Radio-iodinated Anti-VEGFR2 Murine-Human Chimeric Fab. *Sci. Rep.* **5**, 10660; doi: 10.1038/srep10660 (2015).

## Figures and Tables

**Table 1:** Qualification of the necrosis in BEL-7402 xenograft tumors in mice.

**Categorization**	**Level of necrosis**				**χ^2^**	***P***
	−	+	++	+++		
PBS	7	1	0	0	33.253	<0.0001
FA8H1	4	4	0	0		
^131^I	5	2	1	0		
Low dose of ^131^I-FA8H1	0	1	3	4		
Medium dose of ^131^I-FA8H1	0	1	1	6		
Large dose of ^131^I-FA8H1	0	0	2	6		

**Table 2:** The immunohistochemisty data of FasL and Caspase-3 in human HCC xenografts.

	**FasL**				**χ^2^**	***P***	**Caspase-3**				**χ^2^**	***P***
**Categorization**	–	+	++	+++			–	+	++	+++		
PBS	8	0	0	0	27.131	<0.0001	7	1	0	0	31.199	<0.0001
FA8H1	4	3	1	0			3	3	2	0		
^131^I	4	2	2	0			3	3	2	0		
Low dose of ^131^I-FA8H1	0	3	3	2			0	1	2	5		
Medium dose of ^131^I-FA8H1	0	2	3	3			0	1	1	6		
Large dose of ^131^I-FA8H1	0	1	4	3			0	0	2	6		
